# Supplementary material for: Gene Expression Profile of Mexican Lime (Citrus aurantifolia) Trees in Response to Huanglongbing Disease caused by Candidatus Liberibacter asiaticus
Source: Microorganisms. 2020 Apr 7;8(4):528. doi: 10.3390/microorganisms8040528 (PMC7232340; doi:10.3390/microorganisms8040528)
Supplement: Supplementary file 1 [file microorganisms-08-00528-s001.zip › microorganisms-743944-Supplementary Material/microorganisms-743944-Supplementary Figure S1.docx]

Supplementary Materials – Figure S1 and table list

Gene Expression Profile of Mexican Lime (*Citrus aurantifolia*) Trees in Response to Huanglongbing Disease caused by *Candidatus* Liberibacter asiaticus

Ángela Paulina Arce-Leal ^1^, Rocío Bautista ^2^, Edgar Antonio Rodríguez-Negrete ^3^, Miguel Ángel Manzanilla-Ramírez ^5^, José Joaquín Velázquez-Monreal ^5^, María Elena Santos-Cervantes ^1^, Jesús Méndez-Lozano ^1^, Carmen R. Beuzón ^4^, Eduardo R. Bejarano ^4^, Araceli G. Castillo ^4^, M. Gonzalo Claros ^2,6^ and Norma Elena Leyva-López ^1,^*

^1^ Instituto Politécnico Nacional, CIIDIR-Unidad Sinaloa, Departamento de Biotecnología Agrícola, 81101 Guasave, México; [angela_paulina22@hotmail.com](javascript:void(0);) (Á.P.A.-L.); [msantos@ipn.mx](javascript:void(0);) (M.E.S.-C.); [jmendezl@ipn.mx](javascript:void(0);) (J.M.-L.)

^2^ Plataforma Andaluza de Bioinformática, Universidad de Málaga, 29590 Malaga, Spain; [rociobm@uma.es](javascript:void(0);) (R.B.); [claros@uma.es](javascript:void(0);) (M.G.C.)

^3^ CONACyT. Instituto Politécnico Nacional. CIIDIR-Unidad Sinaloa. Departamento de Biotecnología Agrícola, 81101 Guasave, México; [edgarrnegrete@gmail.com](javascript:void(0);)

^4^ Instituto de Hortofruticultura Subtropical y Mediterránea La Mayora (IHSM-UMA-CSIC); Área de Genética, Facultad de Ciencias, Universidad de Málaga, 29010 Málaga, Spain; [cbl@uma.es](javascript:void(0);) (C.R.B.); [edu_rodri@uma.es](javascript:void(0);) (E.R.B.); [ara@uma.es](javascript:void(0);) (A.G.C.)

^5^ Campo Experimental Tecomán-INIFAP. Carretera Colima-Manzanillo km. 35. Tecomán, 28100 Colima, México; [manzanilla.miguel@inifap.com](javascript:void(0);) (M. Á.M.-R.); [velazquez.joaquin@inifap.gob.mx](javascript:void(0);) (J.J.V.-M.)

^6^ Departamento de Biología Molecular y Bioquímica, Universidad de Málaga, 29010 Malaga, Spain

***** Correspondence: neleyval@ipn.mx; Tel.: +52-687-110-0278


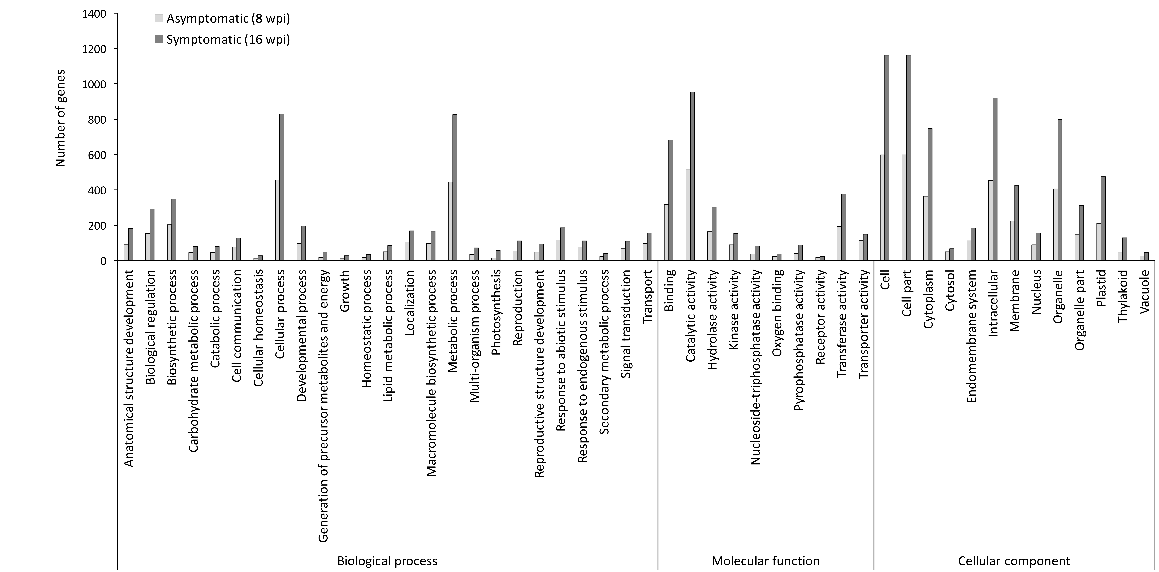


**Figure S1.** Gene ontology analysis of differentially expressed genes in asymptomatic (early) and symptomatic (late) stages of HLB disease.

Supplementary table list

**Table S1.** Primers sequence used for gene expression validation.

**Table S2:** Annotation and expression statistics of differentially expressed genes (DEGs) at asymptomatic and symptomatic HLB disease development stages in Mexican lime.

**Table S3:** Annotation and expression statistics of DEGs related to stress response at asymptomatic and symptomatic HLB disease development stages.
